# Effectiveness of an mHealth Intervention With Short Text Messages to Promote Treatment Adherence Among HIV-Positive Mexican Adults: Randomized Controlled Trial

**DOI:** 10.2196/57540

**Published:** 2025-01-28

**Authors:** Luis Eduardo Del Moral Trinidad, Jaime Federico Andrade Villanueva, Pedro Martínez Ayala, Rodolfo Ismael Cabrera Silva, Melva Guadalupe Herrera Godina, Luz Alicia González-Hernández

**Affiliations:** 1 Public Health Sciences, Universidad de Guadalajara, Guadalajara, Mexico; 2 HIV Unit, Hospital Civil de Guadalajara, Hospital 278, Guadalajara, 44280, Mexico, 52 3338093219; 3 Department of Medical Sciences, University of Guadalajara, Guadalajara, Mexico; 4 Biomedical Sciences Research Institute, Universidad de Guadalajara, Guadalajara, Mexico

**Keywords:** HIV, treatment adherence, mobile health, mHealth, mHealth intervention, randomized clinical trial, text messages, eHealth intervention, sexual health, randomized controlled trial, RCT

## Abstract

**Background:**

HIV continues to be a public health concern in Mexico and Latin America due to an increase in new infections, despite a decrease being observed globally. Treatment adherence is a pillar for achieving viral suppression. It prevents the spread of the disease at a community level and improves the quality and survival of people living with HIV. Thus, it is important to implement strategies to achieve sustained treatment adherence.

**Objective:**

The objective of this study is to evaluate the effectiveness of a mobile health (mHealth) intervention based on SMS text messages to increase antiretroviral therapy (ART) adherence for HIV-positive adults.

**Methods:**

A randomized controlled trial was performed at the Hospital Civil de Guadalajara – Fray Antonio Alcalde on HIV-positive adults who had initiated ART. The mHealth intervention included the use of SMS text messages as a reminder system for upcoming medical examinations and ART resupply to increase adherence. This intervention was provided to 40 participants for a 6-month period. A control group (n=40) received medical attention by the standard protocol used in the hospital. Intervention effectiveness was assessed by quantifying CD4+ T cells and viral load, as well as a self-report of adherence by the patient.

**Results:**

The intervention group had greater adherence to ART than the control group (96% vs 92%; *P*<.001). In addition, the intervention group had better clinical characteristics, including a lower viral load (141 copies/mL vs 2413 copies/mL; *P*<.001) and a trend toward higher CD4+ T cells counts (399 cells/μL vs 290 cells/μL; *P*=.15).

**Conclusions:**

These results show that an mHealth intervention significantly improves ART adherence. Implementing mHealth programs could enhance the commitment of HIV-positive adults to their treatment.

## Introduction

In 2022, 39 million people were living with HIV, of which 37.5 million were over the age of 15 years. Most people living with HIV are female (53%), and 630,000 people died from AIDS-related illness in 2022 [1]. International efforts have been implemented to achieve the 95-95-95 goal by 2030. This goal is that for all people living with HIV, 95% be aware of their HIV status, 95% of all diagnosed people be treated with antiretroviral therapy (ART), and 95% of treated people have an undetectable viral load [2]. HIV continues to be a public health concern in Mexico and Latin America due to an increase in new cases, despite a decrease being observed globally. The high-risk populations in Mexico reflects global trends and include men who have sex with men, injection drug users, sex workers, and transgender individuals. By the end of 2020, there were 3389 new HIV cases reported. Males accounted for 80.2% of cases and females accounted for 19.8%, with a sex ratio of 4:1 [1-4].

Adherence is not only an important pillar for achieving viral suppression, but it also prevents the spread of the virus at a community level. Moreover, viral suppression is a contributing factor for improving the quality of life for people living with HIV [3]. The World Health Organization defines treatment adherence as “the extent to which a person’s behavior, taking medication, following a diet, and/or executing lifestyle changes, corresponds with agreed recommendations from a health care provider” [4,5]. Treatment adherence is complex and is influenced by the personal perceptions that people living with HIV have toward the disease and the relationship they have with health care providers and institutions [6,7]. Multiple factors can impact adherence to treatment, such as the patient’s understanding of the disease, side effects of the treatment, perceived treatment efficacy, trust in health care providers, financial and social access to ART, mental health status, and availability of family or community support [8,9].

Improving treatment adherence is beneficial to the overall health of people living with HIV by reducing disease progression and the development of complications from infection. In addition, treatment adherence is favorable for health systems as it reduces the number of patients with infections, diminishes the need for specialized medical attention, reduces the need for alternative treatments due to treatment failure, and prevents patient disability [10]. Recent research suggests that individuals newly diagnosed with a chronic condition often cultivate their behaviors and perceptions regarding treatment during the initial 6-month period. This time frame is critical for them to establish treatment adherence, which can have far-reaching implications for their long-term health and well-being [11].

Literature has reported a wide range of interventions to improve adherence, including digital interventions (also known as digital health). Digital health is defined as the cost-effective and secure use of information and communication technologies to support health and health-related fields. An integral part of digital health is using wireless technologies like phones, tablets, and smartphones for public health, which is known as mobile health (mHealth) [12]. eHealth and mHealth strategies have been proposed for many aspects of health services, including medication adherence, diagnosis, training, recruitment, follow-up, disease prevention, and guidance about a disease or treatment [13]. In a systematic review analyzing 27 studies regarding the use of mHealth for people living with HIV in low- and middle-income countries, it was shown that 56% of the studies had a significant positive impact on treatment adherence. The most common intervention was the use of reminders through SMS text messages [14].

The duration of interventions can vary depending on the population and objectives of the study, as described in a systematic review by Taylor et al [15] that examined a range of SMS-based interventions across different settings. Additionally, it has been documented that prolonged interventions could potentially lead to participant fatigue and disengagement, which is an important consideration when designing such interventions [16]. One of the main reasons why SMS interventions have been used is due to their extensive coverage in areas where wireless internet is not available. Additionally, SMS text messages can be received by users even if they do not have a data plan. This intervention allows patients to stay connected despite the coverage limitations they may face. Thus, this study aimed to evaluate the efficacy of an mHealth intervention, based on SMS text messages, in increasing ART adherence for adults living with HIV in contrast to standard medical conventions.

## Methods

### Eligibility Criteria

We performed a randomized clinical trial in which we assessed the efficacy of using SMS text messages to improve treatment adherence. People living with HIV were recruited from the HIV clinic of the tertiary care Hospital Civil de Guadalajara – Fray Antonio Alcalde from February to November 2022. The study included both men and women aged 18 years or older who were diagnosed with HIV and had been on ART for less than 6 months. Additionally, participants needed to have a mobile device capable of receiving SMS text messages and sign the informed consent to participate. Excluded from the study were individuals currently enrolled in another clinical trial, pregnant women, minors, and individuals with a mobile phone capable of receiving SMS messages but lacking coverage or service in their residential area.

### Sample Size Determination

The sample size was determined using the OpenEpi software version 3.01, using an anticipated mean difference of 3 percentage points in the rates of adherence between the study groups, based on existing literature [17]. The study was designed with a 95% CI, 80% statistical power, and a 1:1 sample size ratio between the groups being compared. A power analysis was done and determined that a sample size of 29 participants per group was sufficient to detect a mean difference of 3 points in adherence with 80% power and a significance level of .05. Increasing the sample size to 40 participants per group would raise the power to 91%, providing a greater likelihood of detecting a true effect if it exists.

### Randomization

To begin randomization, the protocol nurse generated a table of random numbers in Excel version 16.92 (Microsoft), which was used to assign participants to their respective groups based on whether the randomly generated number was even or odd, at a 1:1 ratio. If a participant who was invited declined the intervention, the allocation sequence proceeded with the next randomly assigned number. This approach ensured that each patient had an equal chance of being allocated to either group, enhancing the randomization process and minimizing selection biases.

### Variables of the Study

Sociodemographic variables were obtained at the time of recruitment using a form that was filled out with the aid of the research team. Viral load and number of CD4+ T cells were measured at the time of enrollment and after the 6-month follow-up. Self-adherence was measured at the 6-month follow-up. To measure adherence, the 4-day adherence index was used, which was calculated using the following formula [18]:


4-day adherence index=1−(forgotten pills/prescribed pills)×100

For the measurement of variables, participants were considered employed if they reported engaging in paid work, whether formal or informal. Tobacco, alcohol, and illicit drug consumption were determined if participants reported consumption more than twice a week in the last month. The duration of time living with HIV and the duration on ART were calculated using the study’s completion date.

### mHealth Strategy

The intervention consisted of the following procedures. Participants received 2 messages per week on Mondays and Thursdays in the morning during the first 3 months, then 1 message per week for the last 3 months. These messages were prevalidated by a research group and categorized into four types: motivational messages for self-care, reminders for ART collection, reminders for medical appointments, and laboratory reminders. The content encouraged patients to maintain adherence to their medication, provided reminders about upcoming medical visits, and reinforced the importance of regular lab tests, such as CD4 and viral load monitoring. To avoid repetition, the messages were rotated throughout the intervention. The full set of validated messages and their detailed content can be found in the corresponding publication [19].

Additionally, participants received reminder messages for appointments or prescription refills 24 hours before the scheduled date during the 6-month intervention period. Moreover, participants had the option to communicate with the researchers to address any queries, which were resolved through a consensus among the research team with support from relevant departments. To ensure ongoing communication, the telephone line accepted collect messages and collect calls, enabling participants to stay connected throughout the study. Both groups received the standard medical treatment provided by the hospital, which consisted of medical appointments tailored to each patient’s needs, but the appointments were scheduled no more than once every 3 months. The treatment also included free ART with a maximum supply of 90 pills. Additionally, laboratory studies and complementary tests were performed in accordance with national treatment guidelines.

### Statistical Analysis

Proportions were analyzed by *χ*^2^ tests and a Student *t* test was used for variables. Relative risk based on treatment adherence (>95% or >90%) was measured and the Fisher exact test was used for statistical evaluation. A multivariate analysis was done to calculate relative risks of variables associated with adherence. All statistical analyses were performed on SPSS version 25 (IBM Corp) and an α level of .05 was used to determine significance.

### Ethical Considerations

The study protocol was approved by the Ethics Committee in Research of the Hospital Civil de Guadalajara – Fray Antonio Alcalde (registry number 095/18) and was registered as a clinical trial (NCT05187741). In line with the Council for International Organizations of Medical Sciences guidelines, all participants provided written inform consent, which was supplemented by a family witnesses in vulnerable cases to ensure fully informed decision-making. The consent form included all relevant information from the protocol and was supplemented with direct clarification of any concerns to ensure patients fully understood. Participants were informed that they could withdraw from the study at any time without any impact on their access to medical care or other services. Privacy was safeguarded by anonymizing participant data with numerical codes, with only the principal research team having access. In the event of unintentional disclosure, a potential risk identified in our study, psychological support channels were made available. While no monetary compensation was provided, participants benefited from direct access to health care professionals via text or call support, and the treatment also included free ART with a maximum supply of 90 pills.

## Results

**Figure 1.**
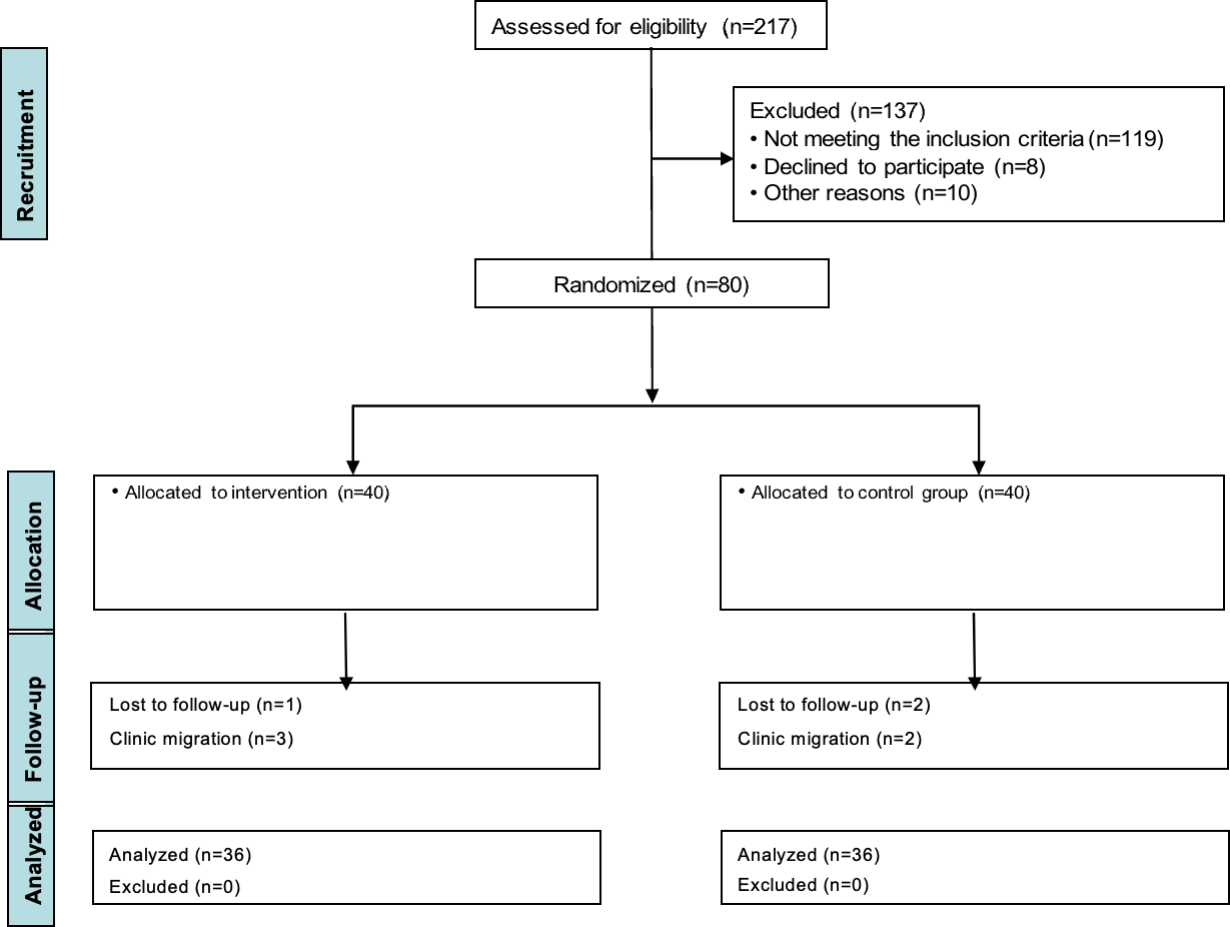
CONSORT (Consolidated Standards of Reporting Trials) flow diagram of the intervention.

From 217 potential candidates, 80 patients fulfilled the inclusion criteria and agreed to participate in the study. The 80 participants were randomly assigned to the intervention or control group (40 in each). In the intervention group, 4 participants were lost to follow-up: 3 due to clinic migrations and 1 for unspecified reasons. In the control group, 2 participants were lost to follow-up due to clinic migrations, and an additional 2 were lost for other reasons. All participants analyzed belonged to their original assigned group (Figure 1). Intervention and control groups had a median age of 31 (IQR 27.8‐36.0) years and 37.5 (IQR 30.0‐49.5) years, respectively. Both groups were mostly men (intervention: 38/40, 96%; control: 37/40, 93%). At the time of enrollment, the intervention group had more CD4+ T cells (227 cells/μL vs 169 cells/μL) and a higher viral load (65,050 copies/mL vs 57,800 copies/mL) compared to the control. In addition, the intervention group had a greater number of days living with HIV and more days on ART when compared to the control group (Table 1). Tobacco consumption was greater in the control group (15/40, 38% vs 23/40, 58%). Most of the participants were single, employed, and reported alcohol consumption (Table 1).

**Table 1.** Participants’ clinical and sociodemographic characteristics.

	Intervention group (n=40)	Control group (n=40)	*P* value
Clinical variables			
Age (years), median (IQR)	31.0 (27.8‐36.0)	37.5 (30.0‐49.5)	.001
Sex, n (%)			—^a^
Male	38 (96)	37 (92)	
Female	2 (4)	3 (8)	
Days after HIV diagnosis, median (IQR)	283.5 (58.3‐519.3)	303 (101.3‐1128)	.50
Days on ART^b^, median (IQR)	122 (15.75‐192.3)	51 (19.75‐171.0)	.73
CD4+ T cell count (cells/μL), median (IQR)	227 (88‐435)	169 (25‐291)	.09
Viral load (copies/mL), median (IQR)	65,050 (17,282-343,750)	57,800 (21,900-168,750)	.46
Sociodemographic variables, n (%)			
Employed	25 (62)	24 (60)	.60
Tobacco consumption	15 (38)	23 (58)	.10
Alcohol consumption	25 (62)	30 (75)	.13
Illegal drug consumption	15 (38)	14 (35)	.81

a Analysis was not performed.

b ART: antiretroviral therapy.

After 6 months, the viral load and CD4+ T cell count were reassessed. The intervention group trended toward higher CD4+ T cell counts compared to the control group (399 cells/μL vs 290 cells/μL; *P*=.15). In addition, the intervention group had a significantly reduced viral load compared to the control group (141 copies/mL vs 2413 copies/mL; *P*<.001). Regarding treatment adherence, the intervention group self-reported a higher level of adherence than the control group (96% vs 92%; *P*<.001; Table 2).

**Table 2.** Clinical characteristics and adherence at the intervention period endpoint.

	Intervention group (n=36)	Control group (n=36)	*P* value
CD4+ T cell count (cells/μL), median (IQR)	399 (238.8‐634.0)	290 (195.3‐557.5)	.15
Viral load (copies/mL), mean (SD)	141 (60)	2413 (1606)	<.001
Adherence rate (%), median (IQR)	96 (94.3‐98.0)	92 (88.0‐95.0)	<.001

We further analyzed adherence and separated the members of each group based on whether they achieved adherence above 90% or 95%. In both cases, the intervention group had a significantly greater chance of reaching the optimal levels of adherence and were approximately 3 times more likely to reach 95% adherence than the control (Table 3).

**Table 3.** Comparison of treatment adherence between the intervention and control groups.

Group	Adherence, n (%)		Relative risk (95% CI)	*P* value
	Yes	No		
Adherence >90%				
Intervention	36 (100)	0 (0)	1.39 (1.31‐1.70)	<.001
Control	26 (72)	10 (28)	1.0 (reference)	—
Adherence >95%				
Intervention	26 (72)	10 (28)	2.6 (1.48‐4.57)	<.001
Control	10 (28)	26 (72)	1.0 (reference)	—

To identify if other variables impacted the probability of reaching 95% adherence, we performed a multivariate analysis. Aside from the SMS intervention, no other variables significantly contributed to reaching 95% adherence (Table 4).

**Table 4.** Multivariate analysis of variables associated with 95% treatment adherence.

Variable	Relative risk (95% CI)		*P* value
Age >45 years	0.73 (0.15‐3.60)		.69
Employed	0.39 (0.10‐1.41)		.15
Tobacco consumption	1.56 (0.436‐5.60)		.49
Alcohol consumption	2.31 (0.59‐8.98)		.22
Use of illegal drugs	0.81 (0.25‐2.62)		.73
Detectable viral load	4.26 (0.67‐26.83)		.12
CD4+ T cell count >200 cells/μL	4.09 (0.38‐43.05)		.24
Study intervention	11.66 (2.88‐47.05)		.001

## Discussion

### Principal Findings

This study aimed to evaluate the effectiveness of an mHealth intervention on adherence for adults starting ART. As previously mentioned, ART adherence is important for people living with HIV, allowing them to reach viral suppression and increasing their overall quality of life. This highlights the importance of exploring interventions to support patients recently diagnosed with HIV to reach optimal adherence. This study enrolled patients with an age ranging from 30 to 40, which corresponds with the national and worldwide age with the highest HIV prevalence. Also, our participants were mostly men, which aligns with Mexico’s national trend, as men who have sex with men and transgender people are the main groups affected by HIV in Mexico [20].

Both the intervention and control groups shared previously reported factors that can impact adherence like alcohol consumption and being single [5,6,10,21]. Although further analysis of the factors associated with adherence was beyond the scope of this study, our descriptive results provide a preliminary view for further investigations aiming to study the importance of these factors on a patient’s adherence to treatment. Even though the participants were randomly assigned to each group, our intervention group was younger than our control group. We believed that this was related to the recruitment process, as older patients were more likely to reject the invitation of the study due to the concern of using digital conventions over in-person conventions. Giebel et al [22] mention that interventions must be adapted to different age groups due to the diversity of digital health users. These findings need to be explored and contemplated in further studies.

Regarding our clinical findings, the control group had a lower CD4+ T cell count (168 cells/μL), suggesting a delay in HIV detection. The fact that one of our study groups had less than 200 cells/μL highlights the relevance of finding methods to improve early detection in high-risk populations and strategies to prevent low adherence to ART or losses to follow-up [23]. One of the potential implications of these findings is the application of mHealth interventions to provide a faster linkage-to-care after HIV diagnosis, as a previous study has shown that these interventions can reduce the gap between health care clients and institutions to increase retention in care and impact mortality [24].

In this study, we show that mHealth strategies improve adherence to treatment by 4% (*P*<.001) and increase the probability of reaching a 95% of treatment adherence by 3 times. However, we wanted to evaluate the impact of age and other variables on the probability of reaching 95% treatment adherence. For this, we performed a multivariate analysis, in which the mHealth intervention statistically correlated with ART adherence regardless of other independent variables like age, marital status, and employment. These results suggest that mHealth strategies can be beneficial in multiple scenarios.

As previously demonstrated, simply sending SMS text messages had minimal to no impact on enhancing treatment adherence [25,26]. Therefore, we implemented an mHealth strategy based on the use of interactive SMS text messages, in which the patients had the opportunity to interact by text or phone call with a health professional. Interactive SMS text messages are effective and participants are more likely to engage actively and adhere to their treatment. [27-29]. Another key aspect of the intervention was the frequency of the text messages. The goal was to help establish a habit in the patients while still providing access to assistance if needed. Also, it has been reported that a high frequency of messages over an extended period can become repetitive and lose their beneficial impact [30-32].

Although other studies have used similar strategies, this is the first study in the Mexican population to use an mHealth approach that decreased message frequency to enhance adherence. In addition, we implemented messages that were validated by a Mexican group of health professionals, researchers, and people living with HIV on ART [19]. Few studies looking into the efficacy of mHealth SMS interventions use validated messages for their population [33-35]. Like other studies, we identified an improvement in treatment adherence by using SMS text messages [14,26,36,37]. However, it is important to consider that adherence evaluation was mainly assessed by a self-report that was provided by the patient, which had an intrinsic bias. As a method to address this bias, we measured the HIV viral load along with adherence reported by the 4-day adherence index, which has been reported to have an adequate sensitivity and specificity in Mexican populations [18]. This allowed us to provide a more accurate estimation of adherence levels, as self-reported measures are known to overestimate true adherence levels.

Although we identified an increased adherence using an mHealth strategy, further studies on a larger population are required to confirm our results. It is also important to recognize the variability among different populations and regions [38]. This study was performed in the State of Jalisco, and we found a 93% treatment adherence rate in our control group. This result was similar to another study performed in the state of Mexico that had a 93% adherence rate [39] but double that of a study done in the state of Tamaulipas that had a 46% adherence rate [40]. These state-specific differences highlight how treatment adherence is contextual and is determined largely by the conditions surrounding the patients [41].

This study had some limitations. The evaluation period was relatively brief, lasting only 6 months, which raised uncertainty about the long-term persistence of the intervention’s effect. Additionally, the reliance on self-reported adherence data, without the use of more objective measures such as pill counts at each appointment, introduced the potential for bias. However, the investigators ensured that all participants underwent viral load testing after the study, providing an objective assessment of treatment outcomes.

### Conclusions

In summary, this study demonstrates that integrating mHealth strategies as a supplementary approach to existing interventions can enhance treatment adherence among people living with HIV. Additionally, our findings highlight significant results that may inform the implementation of larger and more intricate interventions aimed at bridging the communication gap between health care clients and medical service providers.

This kind of mHealth-based intervention could serve as a precursor for larger-scale interventions aimed at increasing patient adherence and retention for chronic illnesses, which is a significant public health concern that we must address. By leveraging digital health technologies, we can develop more comprehensive strategies to support patients in managing their conditions and improve overall treatment outcomes. Such approaches have the potential to enhance communication between health care providers and clients, thereby promoting better treatment adherence and increasing retention rates across diverse populations with chronic diseases.

## Supplementary material

10.2196/57540 Checklist 1 CONSORT-EHEALTH (Consolidated Standards of Reporting Trials of Electronic and Mobile Health Applications and Online Telehealth) checklist (version 1.6.1).

## Abbreviations

ART: antiretroviral therapy
mHealth: mobile health
